# Evidence for anticipation in Beckwith–Wiedemann syndrome

**DOI:** 10.1038/ejhg.2013.71

**Published:** 2013-04-10

**Authors:** Siren Berland, Mia Appelbäck, Ove Bruland, Jasmin Beygo, Karin Buiting, Deborah J G Mackay, I Karen Temple, Gunnar Houge

**Affiliations:** 1 Center for Medical Genetics and Molecular Medicine, Haukeland University Hospital, Bergen, Norway; 2 Department of Clinical medicine, University of Bergen, Bergen, Norway; 3 Institut für Humangenetik, Universitätsklinikum Essen, Essen, Germany; 4 Faculty of Medicine, University of Southampton, Southampton, UK; 5 Wessex Genetics Service, Southampton University Hospitals Trust, Southampton, UK; 6 Salisbury Hospital NHS Foundation Trust, Salisbury, UK

**Keywords:** Beckwith–Wiedemann syndrome, anticipation, imprinting, *H19*, *IGF2*

## Abstract

Classical Beckwith–Wiedemann syndrome (BWS) was diagnosed in two sisters and their male cousin. The children's mothers and a third sister were tall statured (178, 185 and 187 cm) and one had mild BWS features as a child. Their parents had average heights of 173 cm (mother) and 180 cm (father). This second generation tall stature and third generation BWS correlated with increased methylation of the maternal *H19/IGF2*-locus. The results were obtained by bisulphite treatment and subclone Sanger sequencing or next generation sequencing to quantitate the degree of CpG-methylation on three locations: the *H19* promoter region and two CTCF binding sites in the *H19* imprinting control region (ICR1), specifically in ICR1 repeats B1 and B7. Upon ICR1 copy number analysis and sequencing, the same maternal point variant NCBI36:11:g.1979595T>C that had been described previously as a cause of BWS in three brothers, was found. As expected, this point variant was on the paternal allele in the non-affected grandmother. This nucleotide variant has been shown to affect OCTamer-binding transcription factor-4 (OCT4) binding, which may be necessary for maintaining the unmethylated state of the maternal allele. Our data extend these findings by showing that the OCT4 binding site mutation caused incomplete switching from paternal to maternal ICR1 methylation imprint, and that upon further maternal transmission, methylation of the incompletely demethylated variant ICR1 allele was further increased. This suggests that maternal and paternal ICR1 alleles are treated differentially in the female germline, and only the paternal allele appears to be capable of demethylation.

## Introduction

Beckwith–Wiedemann syndrome (BWS, OMIM no. 130650) is an overgrowth condition caused by epigenetic or genetic alterations in the imprinted *H19/IGF2-KCNQ1/CDKN1C* locus, spanning nearly 1 Mb in 11p15.5.^1^ The two major causes of BWS are increased *IGF2* expression or decreased *CDKN1C* expression. The growth inhibitor *CDKN1C* is normally expressed from the maternal chromosome 11. On the paternal chromosome, inhibition of *CDKN1C* is associated with the expression of a long noncoding RNA called *KCNQ1OT1* or *LIT1* antisense to *KCNQ1*, the potassium channel gene involved in long-QT-syndrome type 1 and Jervell/Lange-Nielsen syndrome. In contrast, the growth factor *IGF2* gene is expressed from the paternal chromosome only. On the maternal chromosome, the noncoding RNA gene *H19* is expressed instead. The choices between *H19* or *IGF2* expression, and *LIT1* or *CDKN1C* expression, are regulated epigenetically. *IGF2* expression is associated with methylation of the insulator (CTCF) binding sites between *H19* and *IGF2*, also called imprinting centre region 1 (ICR1), and *CDKN1C* expression is associated with methylation of the *LIT1* promoter, also called imprinting centre region 2 (ICR2).

The regulation of 11p15.5 imprinting that causes monoallelic paternal *IGF2* expression and monoallelic maternal *CDKN1C* expression is complex, which also explains why there are many different molecular causes of BWS.^2^ The most common cause is reduced expression of *CDKN1C*, which is usually due to sporadically occurring reduction in maternal ICR2 methylation (∼50% of BWS cases), but sometimes associated with maternal *CDKN1C* mutations (5–10%). The second most common cause is *IGF2* overexpression, usually due to paternal uniparental disomy of 11p (∼20% of BWS cases), but also to inappropriate ICR1 methylation on the maternal allele, which inhibits *H19* and stimulates *IGF2* expression (∼5%).^1^ In the latter situation, small deletions or mutations in the ICR1 that are likely to disrupt the insulator function of the region have been observed with a high sibling recurrence risk.^3, 4, 5, 6, 7, 8^ Recently, it was found that such small and overlapping deletions of ICR1 had variable effects on methylation of the maternal ICR1, indicating that maintenance of maternal hypomethylation was partly dependent on the spatial arrangement of the CTCF binding sites.^8^

Here, we describe a family with a previously reported ICR1 single nucleotide variant (NCBI36:11:g.1979595T>C) in an OCTamer-binding transcription factor-4 (OCT4) binding site and a gradual increase in ICR1 methylation over the next two generations, the first generation being tall statured, the second generation having full-BWS phenotype with Wilms tumours. This family indicates that ICR1 mutations may affect the ability to establish a maternal ICR1 methylation pattern of the paternal allele in female gonads, that is, to demethylate the paternal ICR1 region. Our data also indicate that the maternal and paternal ICR1 alleles are treated differently in the maternal gonads, that is, that the maternal alleles are not demethylated (if methylated) in the maternal germ line, and may later be subject to a passive (stochastic) increase in methylation. To the best of our knowledge, this is the first description of anticipation in an epigenetic syndrome.

## Patients and Methods

### Family

**Figure 1:**
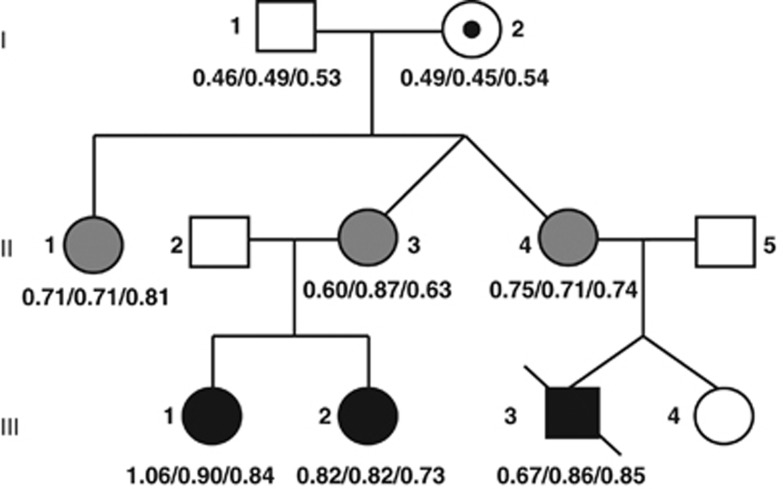
Family pedigree. The degree of methylation of the locus between *H19* and ICR1 was investigated by routine MLPA testing and bisulphite subclone sequencing, indicated by the (first ratio/) and (/middle ratio/), respectively. The methylation of the CTCF binding site 6 (CTS6) within the B1 repeat was investigated by bisulphite next generation sequencing, indicated by the (/final ratio) below the pedigree symbols.

BWS was diagnosed in two sisters (III-1 and III-2) and their male cousin (III-3, see Figure 1). Elective caesarean section was performed in both sisters due to large babies. Both sisters had classical BWS features including Wilms tumour and visceromegaly. III-1 was born in week 38 with macroglossia and large kidneys, birth weight was 4860 g (860 g>97.5^th^ centile), length 53 cm (97.5^th^ centile). As an infant, she was successfully treated for Wilms tumour with chemotherapy. At age 6 years, an operative tongue reduction was performed. III-2 was born at term with macroglossia and large kidneys, birth weight 5280 g (880 g>97.5^th^ centile). At age 9 months, she was nephrectomised due to Wilms tumour in her right kidney and mild nephroblastomatosis in her left kidney was also detected. The sisters are now 10 and 13 years, and both have good school performances and growth parameters in upper percentiles (III-1 97.5^th^ centile and III-2 95^th^ centile). Their male cousin (III-3, DZ twin) died from medical complications after a caesarean section in week 29. There was marked polyhydramnios. Birth weight was 2130 g (330 g>97.5^th^ centile), length 44 cm, head circumference 28 cm and he had visceromegaly (especially of the kidneys), macroglossia and general subcutaneous oedema. In comparison, his healthy unaffected DZ sister III-4 was 1170 g (5^th^ centile), 38 cm and had the same head circumference at birth. None of the three affected children had neonatal hypoglycaemia, were markedly asymmetric or had transverse creases on their ear helices. The children's two mothers and the mothers' sister were tall statured (178, 185 and 187 cm, that is, lengths from the 96^th^ centile and above) with large hands, and at least one had mild BWS features (large tongue, protruding stomach) as a child (II-1 in Figure 1). The sisters' parents had heights of 173 cm (mother, 85^th^ centile) and 180 cm (father, 75^th^ centile), and none of them had any BWS-like features as infants. II-1 was born at term with a large tongue and birth weight 5 kg (0.4 kg>97.5^th^ centile). II-3 and II-4 are DZ twins born in week 31 with birth weights 1800 g (50^th^ centile) and 2350 g (99^th^ centile), respectively, and II-4 also had a large tongue. All sisters have very mild and asymptomatic scoliosis, no hemihyperplasia and normally sized tongues as adults.

### Methylation specific multiplex ligation-dependent probe amplification (MS-MLPA) analysis and bisulphite treatment followed by subclone sequencing

Blood DNA samples were obtained from all individuals except III-1 and III-2, where only saliva DNA samples could be obtained. MS-MLPA copy number and methylation analysis of the BWS/Silver-Russell syndrome region on chromosome 11 was done in the routine diagnostic laboratory using the SALSA MLPA ME030 kit version C2 (MRC-Holland, Amsterdam) and following the manufacturer's instructions. We also obtained saliva DNA samples from II-1 and II-4 to compare with the results of MS-MLPA analysis of blood DNA, and similar levels of methylation was found: 0.7 in saliva DNA from both individuals, compared with 0.71 and 0.75 in their blood DNA (Figure 1). The bisulphite conversion of DNA was performed with Applied Biosystems methylSEQr Bisulphite Conversion Kit (Life Technologies, Carlsbad, CA, USA), according to the manufacturer's protocol. PCR was performed using primers designed by Methyl Primer Express Software v1.0 (Applied Biosystems, Life Technologies) for bisulphite sequencing-specific PCR. Forward primer: 5′-ATTATTTTGGTTTTTGGTGAGG-3′ (unconverted: 5′-ACCACCTTGGCCTTTGGTGAGG-3′); reverse primer: 5′-ATACCATAAAAATTCCCCCATA-3′ (unconverted: 5′-ATGCCATGGAAATTCCCCCATG-3′) (HG19: chr11:2019867-2020153). M13 tails were added to all the primers as part of our routine to obtain uniform PCR conditions. PCR was performed in a 25 μl reaction containing 1x AmpliTaq Gold 360 Master Mix Forward (Invitrogen, Life Technologies, Carlsbad, CA, USA), 16% 360 CG enhancer (Invitrogen, Life Technologies), 10 μM forward primer and 10 μM reverse primer. The PCR conditions were as follows: denaturation at 95 °C for 5 min, 5 cycles of 95 °C for 30 s, 50 °C for 2 min and 72 °C for 3 min, followed by 35 cycles of 95 °C for 30 s, 58 °C for 1 min and 72 °C for 3 min, and finally a hold at 60 °C for 60 min. The PCR products were then cloned using TOPO TA Cloning Kit for Sequencing (Invitrogen, Life Technologies), following the manufacturer's instructions. Ten clones were purified using QIAprep Spin Miniprep kit (Qiagen, Hilden, Germany) and sequenced by Sanger sequencing using the BigDye Terminator v1.1 Cycle Sequencing Kit (Applied Biosystems, Life Technologies, Calrsbad, CA, USA) with T3 or T7 primers. Clean up was performed using the using Big DyeX Terminator purification (Applied Biosystems, Life Technologies). The sequences were resolved on a 3730 Genetic Analyser (Applied Biosystems, Life Technologies) and analysed with QUMA (Riken, Japan). To calculate the average methylation level in controls (*n*=5, including the grandmother of the family) and affected (*n*=3 per generation, see Figure 1), the average of all measurements per sample was first calculated, and thereafter the mean per group (Table 1). For MS-MLPA, four repeated measurements per sample were done, and for bisulphite sequencing, seven informative positions with differential methylation were measured once per patient (see Supplementary Table 1 and Supplementary Figure 1 with legend for details). Five data points were noninformative because they were highly methylated in all samples with no difference between patients and controls (Supplementary Table 1), and these data points were therefore excluded. The individual MS-MLPA and bisulphite data can also be seen in Figure 1, and mean data with 95% confidence intervals can be found in Table 1.

### Bisulphite treatment followed by next generation sequencing

Bisulphite treatment was conducted using the EZ DNA Methylation-Gold Kit (Zymo Research Europe, Freiberg, Germany) according to the manufacturer's manual. For each individual bisulphite amplicon libraries were generated and sample-specific barcode sequences were added. The amplicons were purified, diluted and clonally amplified in an emulsion PCR before sequencing on the Roche/454 GS junior system was carried out. For subsequent data analysis the Geneious software (Biomatters, Auckland, New Zealand) and BiqAnalyzer HT were used.^9^ A detailed description has been published elsewhere.^8^ A minimum of 1058 reads for each sample was obtained. The average conversion rate was 99.0% for CTS1 and 99.1% for CTS6.

### ICR1 sequencing

After confirmation of complete 11p15 grandmaternal haplotype segregation with large growth/BWS (Supplementary Figure 2), ICR1 and the H19 proximal promoter (HG19:chr11:2,020,402-2,024,682) were sequenced by standard methods from a product of 4281 bp generated using primers 5′-TGCACATACTTTGCACATGG/CGCTGTGGCTGATGTGTAG-3′, as described;^3^ further details are available on request. To determine the parental origin of the sequence variant, 200 ng genomic DNA was cleaved using restriction enzyme McrBc (New England Biolabs, Ipswich, MA, USA) following the manufacturer's instructions; then DNA was desalted and concentrated using Amicon 30K microconcentrator columns (Millipore, Billerica, MA, USA) before amplification using primers 5′-CAACACAAGGATCCTAGACC/TCTTCGTATCGGGCCATATC-3′ and Sanger sequencing.

## Results

**Figure 2:**
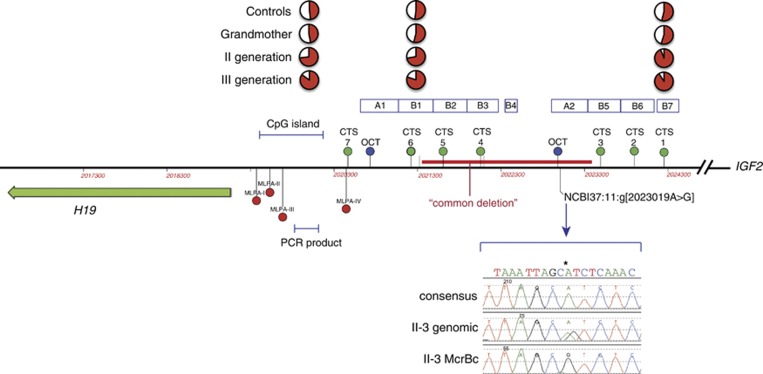
Illustration of the H19/IGF2 ICR1. Above the line approximate positions of CTCF binding sites are marked with green dots, and OCT4 sites are marked with blue dots. The position of a CpG island in the *H19* promoter is shown above the line. Below the line the differentially methylated CpG sites investigated by the routine MLPA kit are marked with red dots, and these sites are not included in the segment from the CpG island (marked as ‘PCR product') that was investigated by bisulphite treatment followed by subclone sequencing and that contained seven differentially methylated CpG sites. The position of the common ∼1.8 kb microdeletion^4^ associated with BWS is marked with a red bar, and the OCT4 binding site mutation is also shown, as well as the sequencing result of II-3 before and after McrBc digestion of methylated DNA. Please note that the g.2023019A>G mutation corresponds to the T>C mutation on the antisense strand, which was described by^10^. The position of CTCF binding sites (CTS) in ICR1 are shown, and of these CTS6 and CTS1 were investigated by next generation bisulphite sequencing.

Our family shows dominant inheritance of BWS with a clinical picture that is compatible with anticipation (Figure 1). This impression was in line with the results of the routine MS-MLPA methylation testing of the BWS locus, which showed an increase in ICR1 methylation from normal level (0.49) in the grandmother to ∼0.71 in the second generation with tall stature and ∼0.84 in the third generation (Table 1, Supplementary Table 1), in the three children with classical BWS (Figure 1). These data were reproduced by bisulphite treatment and subclone sequencing to measure the degree of CpG-methylation of the *H19* differentially methylated promotor region (Figure 2).

**Figure 3:**
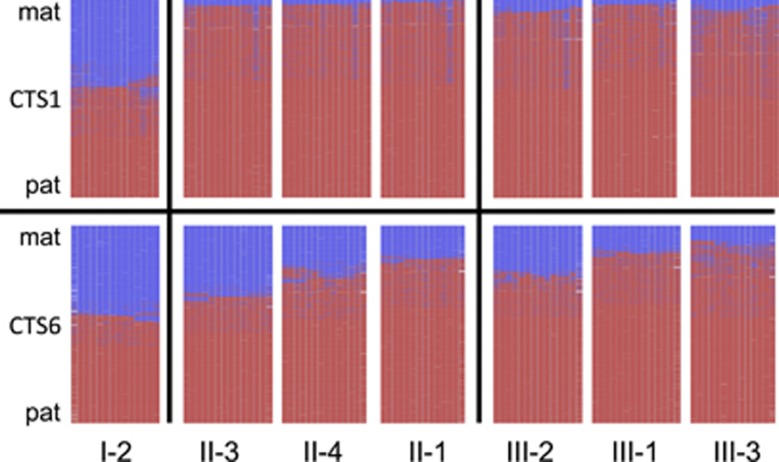
DNA methylation analysis: Heat maps of the methylation patterns obtained by next generation bisulphite sequencing of two CTCF binding sites: CTS1 and CTS6. The heat maps are ordered from left to right by the degree of methylation, and this also corresponds to the clinical severity. Only data from individuals harbouring the allele with the OCT4 binding site mutation is shown. The pedigree marks below the panels are the same as in Figure 1. Lines represent sequence reads, columns CpGs. Blue – unmethylated – maternal (mat); red – methylated – paternal (pat); white – missing sequence information.

To investigate whether the same tendency could be found in the CTCF binding sites (called CTS and numbered from 1 to 7) of the ICR1 repeats (Figure 2), the degree of methylation of two such sites was investigated by highly quantitative next generation bisulphite sequencing: CTS6 in B-type repeat B1, on the *H19*-side of ICR1, and CTS1 in B-type repeat B7, on the *IGF2*-side of ICR1 (Figure 2). Although CTS1 was fully methylated already in the grandmother's children (II-1, II-3 and II-4; Supplementary Table 1), CTS6 showed the same tendency towards increased methylation from generation II to III. In the mothers of generation II, the average degree of methylation was 73%, and in their children, the average degree of methylation was 81% (Figure 1). It thus seems that CTS1 was more prone to acquire methylation than CTS6.^8^ To further illustrate the molecular correlation to the observed clinical anticipation, methylation heat maps of the degree of CTS1 and CTS6 methylation from the grandmother to daughters to grandchildren are shown in Figure 3. Of note, the degree of methylation in CTS6 correlates well with the clinical severity (Figure 3), that is, the number of BWS symptoms and findings (see above; II-3<II-4<II-1 and III-2<III-1<III-3). All normal individuals had heat map plots showing average (∼50%) methylation (Supplementary Figure 3).

As dominant BWS can be associated with mutations in ICR1, between the *H19* noncoding RNA gene and the growth factor gene *IGF2*, this region was examined for mutations or deletions. Long-range PCR did not detect any microdeletions in ICR1 that could explain imprinting disturbances. However, ICR1 sequencing revealed the same maternal point variant NCBI36:11:g.1979595T>C (NCBI37:11:g.2023019T>C, see Figure 2) that had been described previously as a cause of BWS in three brothers.^10^ To determine the parental origin of the variant, genomic DNA was digested with the methylation-sensitive restriction enzyme McrBC, which digests methylcytosine-containing DNA, and then amplified and sequenced. In the digested DNA from II-1 and II-3, the variant became apparently homozygous (II-3 result shown in Figure 2), indicating that it was present on the partially unmethylated, and therefore maternally inherited allele. In I-2, the non-affected grandmother, the opposite was found, indicating that the point variant was on the grandmother's paternal allele. Haplotype and sequencing information showed that the three sisters in generation II and the three affected children in generation III inherited this point variant, but not by the unaffected DZ sibling in generation III (Supplementary Figure 2).

## Discussion

From a clinical point of view, the second generation tall stature and third generation BWS in our family suggested anticipation (Figure 1). Alternatively, the increased clinical severity might be a random result of variable expression, not uncommon in BWS (see e.g. Scott *et al.*^11^). However, the increasing degree of methylation from the unaffected carrier grandmother of a paternal ICR1 variant over the two subsequent generations gave a molecular correlate to the observed anticipation (Figures 1 and 3, Table 1). The increased degree of ICR1 methylation was found both on routine MLPA-based methylation testing and bisulphite subclone sequencing of the CpG island region in the *H19* promoter, and bisulphite next generation sequencing of the CTCF binding site in the B1 repeat region of ICR1 (CTS6), but not the more *IGF2*-proximal CTCF binding site in the B7 repeat (CTS1) (Figures 2 and 3). Upon ICR1 sequencing to find a cause of the apparent anticipation, a previously found variant affecting OCT4 binding was found (Figure 2).^10^ It was highly unlikely that this represented a founder mutation as the other family was French and the Norwegian family had no known French roots. Nevertheless, to exclude that this could be a founder mutation, Christine Gicquel was most helpful and sent us the SNP information of the ICR1 locus in their family, and when compared to our family, a common haplotype was excluded. We could therefore conclude that the same NCBI37:11:g.2323019T>C (NCBI36:11:1979595T>C) variant had occurred on two different genetic backgrounds.

Recently, a family with another variant reducing OCT4 binding was described.^3^ This variant 1979624A>C was 31 nt centomeric to the 1979595T>C variant found by Demars *et al.*^10^ and us. Of note, in this family there were two affected brothers with prolonged post pubertal growth and final heights above the 99^th^ centiles (204 cm and 208 cm, respectively), which is atypical for BWS patients. They also had renal problems, one had cysts and the other had a Wilms tumour, and neither was markedly asymmetric. This suggests that there may be clinical features that distinguish ICR1 mutation patients from other patients with BWS. None of the reported patients had the characteristic ear creases and growth did not decelerate in their teens, resulting in final statures above the 97.5^th^ centile. If the latter also will hold true for the third generation in our family is yet unknown, as they are all children. Nevertheless, if a child with BWS and ICR1 hypermethylation has a tall statured mother, this should alert to the possibility of dominant inheritance, especially if the child also has Wilms tumour. Absence of ear creases should strengthen this suspicion.

It has previously been shown that the T>C variant in the ICR region affects OCT4/SOX2 binding,^10^ and our data can be interpreted to imply that the variant interferes with gonadal switching from paternal to maternal imprinting. The same may be true for the A>C variant reported by Poole *et al.*^3^ In the latter case, however, classical BWS occurred already in the first generation. One explanation for this discrepancy could be that the A>C variant is more detrimental to OCT4 binding than the T>C variant. Alternatively, the cause could be random variability in clinical expression. Our family suggests, however, that true anticipation as an explanation for increased clinical severity may take place in BWS. This is supported by a recent report on the consequences of ICR1 microdeletions, where at least two of the six families had features compatible with anticipation, described as ‘a kind of epigenetic memory effect' by the authors.^8^ Possibly, the phenomenon of anticipation in BWS is not limited to OCT4 binding site mutations, but could be an explanation for non-penetrance in ICR1 deletion families as well. This has implications for genetic counselling of BWS families with ICR1 deletions or mutations. Transmission through the male germline implies a potential BWS risk not necessarily in the next, but in subsequent generations if the mutation is passed through the female germline. Second, transmission of the mutation through the maternal germline may first give rise to BWS in later (third and fourth) generations if the mutation is ‘mild'.

From a more fundamental biological perspective, our findings give clues to how gonadal imprinting switching is regulated. The grandmother inherited a fully methylated paternal allele containing the T>C variant from her father, and a normal demethylated maternal allele from her mother. In the next generation, her three daughters inherited the grandpaternal ICR1 allele (with the T>C variant) and they had methylation levels of around 73%. This implies either that the grandpaternal allele was incompletely demethylated in their mother's gonads or that the variant allele was completely demethylated in the germline, but had reduced resistance to subsequent postzygotic remethylation. There are experimental data to support both of these suggestions.^12^ A recent study showed that interference with OCT4 binding in P19 embryonic carcinoma cells rendered the H19 allele partially resistant to demethylation, but also that when partially methylated, further methylation seeding (remethylation) could easily take place.^13^ Methylation seeding implies that inappropriate methyl groups recruit binding proteins that directly and indirectly promote both histone compaction and further DNA methylation. Moreover, on a more general genomic scale it has been shown that maintenance of imprinting marks in early zygotic development requires not only protection against postfertilisation demethylation, but also protection against somatic remethylation.^14^ A similar requirement for OCT4/SOX2-binding elements to maintain the maternal demethylated state has been found in the Prader-Willi/Angelman syndrome imprinting centre.^15^

**Figure 4:**
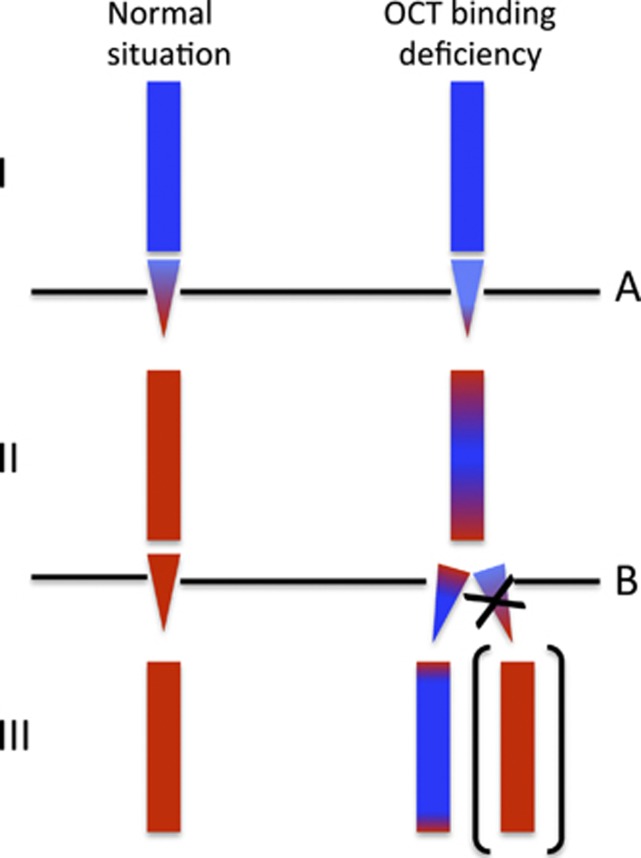
The imprinting status of the grand-grand-paternal allele after grandmaternal and maternal transmissions. The OCT4 binding site mutation apparently causes incomplete demethylation upon grandmaternal transmission (A), and further methylation upon maternal transmission (B). Of note, further demethylation, as in A, was not seen in B (marked by the X), suggesting that only paternal alleles can be demethylated in the maternal gonads. Blue=methylated (paternal pattern) and red=unmethylated (maternal pattern).

To explain anticipation, reduced protection against somatic remethylation is not enough. There must also be an element of demethylation resistance in the germline. However, even if the mutation caused only partial ICR1 demethylation to take place in the female germline (grandmother and daughters), one would still expect less ICR1 methylation in third generation (the children with BWS), not more. This remains true even if a susceptibility to somatic remethylation should vary somewhat from individual to individual. Of note, the grandpaternal (normal) allele inherited by III-4 (also confirmed by haplotyping, see Supplementary Figure 2) was completely demethylated in the same maternal gonad that could not demethylate the grandmaternal allele (II-4 in Figure 1). This indicates that mutations interfering with OCT4 binding may have different effects in paternal and maternal inheritance. Apparently, unlike the paternal allele, the maternal allele is incapable of being actively demethylated in the maternal germline—otherwise there would have been no anticipation. Our hypothesis on differential handling of paternal and maternal alleles in the maternal gonads is illustrated in Figure 4.

In conclusion, we have both clinical and molecular evidence for the occurrence of anticipation in rare cases of BWS, and our data also indicate that only paternally inherited *H19/IGF2* loci are demethylated in the maternal germline.

## Figures and Tables

**Table 1:** Mean degree of ICR1 methylation in the first, second and third generations with 95% confidence intervals (parentheses)

	MLPA-test	Bisulphite sequencing
1. generation (*n*=2)	0.48 (0.45–0.51)	0.47 (0.39–0.55)
2. generation (*n*=3)	0.71 (0.66–0.76)	0.75 (0.72–0.78)
3. generation (*n*=3)	0.84 (0.81–0.87)	0.86 (0.81–0.91)

Abbreviations: ICR1, imprinting control region; MLPA, multiplex ligation-dependent probe amplification.
