# Supplementary figures and images for: Evidence for anticipation in Beckwith–Wiedemann syndrome
Source: Eur J Hum Genet. 2013 Apr 10;21(12):1344–8. doi: 10.1038/ejhg.2013.71 (PMC3831082; doi:10.1038/ejhg.2013.71)

|       | incl                                                                               | excl                                                                               |
|-------|------------------------------------------------------------------------------------|------------------------------------------------------------------------------------|
| I-2   | 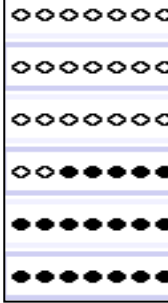  | 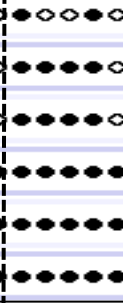  |
| II-4  | 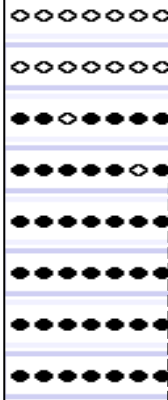  | 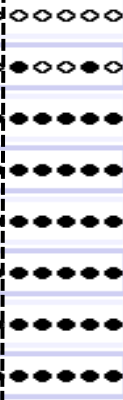  |
| III-3 | 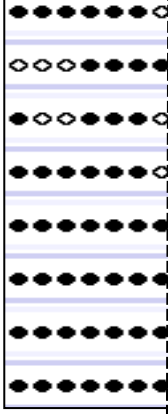 | 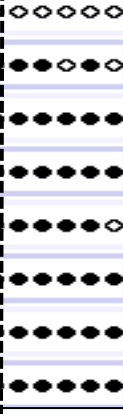 |

Supplement: Supplementary Figure 1 [file ejhg201371x1.pdf]

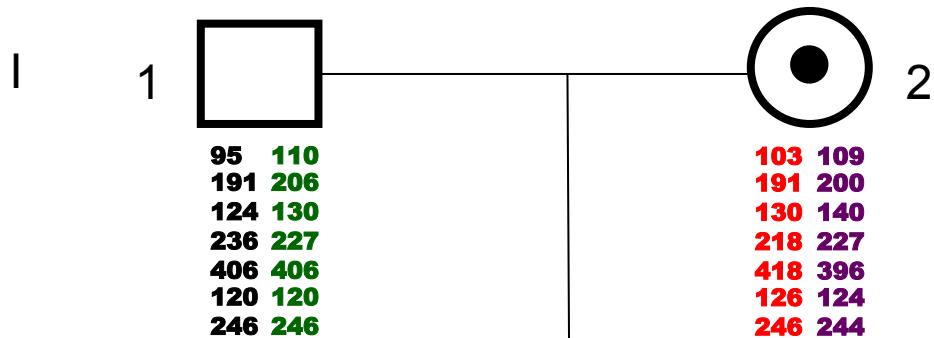

|            | Markers  | Genomic pos |
|------------|----------|-------------|
|            | D11S922  | 1605090     |
|            | D11S4046 | 1963642     |
| H19/IGF2 → | D11S1318 | 2327223     |
| CDKN1C →   | D11S4088 | 2754951     |
|            | D11S1923 | 3027720     |
|            | D11S988  | 4539851     |
|            | D11S1758 | 4735970     |

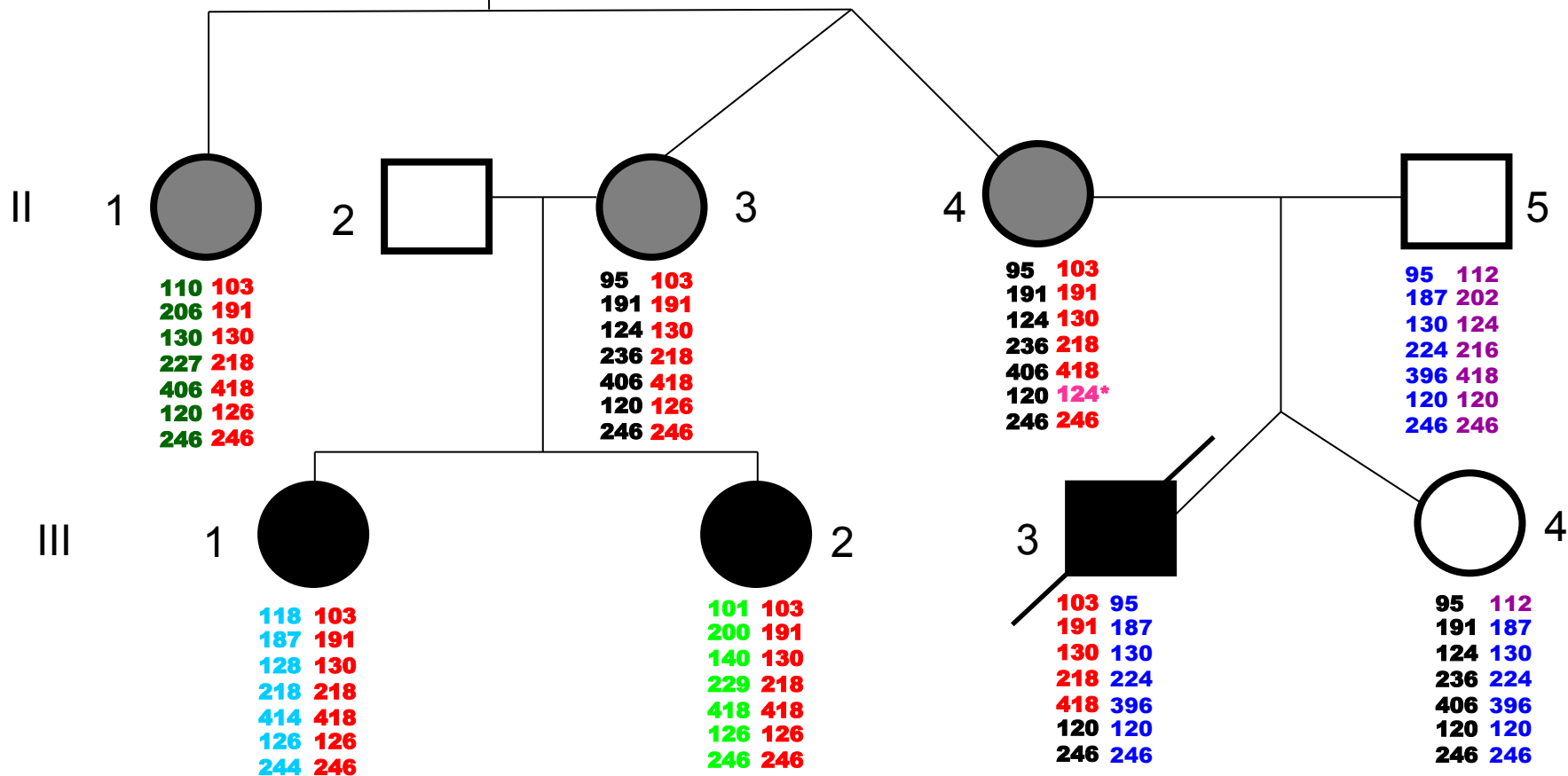

Supplement: Supplementary Figure 2 [file ejhg201371x2.pdf]

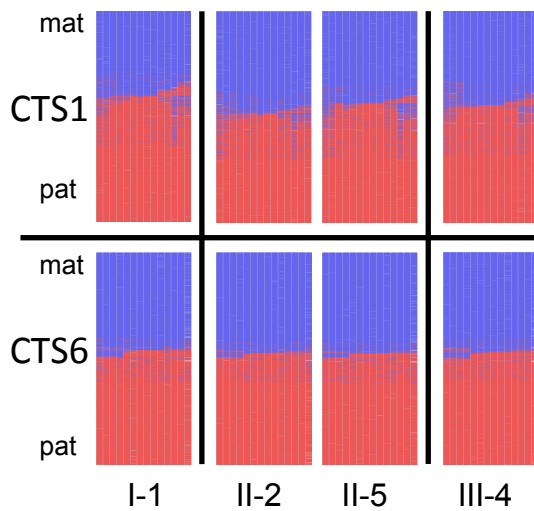

Supplement: Supplementary Figure 3 [file ejhg201371x3.pdf]
