# Supplementary material for: Evidence for anticipation in Beckwith–Wiedemann syndrome
Source: Eur J Hum Genet. 2013 Apr 10;21(12):1344–8. doi: 10.1038/ejhg.2013.71 (PMC3831082; doi:10.1038/ejhg.2013.71)
Supplement: Supplementary Figure Legends [file ejhg201371x4.doc]

**Supplementary Figure 1:** This figure shows how the degree of methylation of an ICR1 segment (see Figure 2 for location) was measured by cloning and bisulphite sequencing, exemplified by three family members: I­2, II-4 and III-3. The segment contained 7 differentially methylated CpGs (the seven first positions) and 5 non-informative CpG positions that were excluded because of no differences between patients and controls, and stably high methylation degrees of 75-93%. The CpG positions are represented by circles, and a filled circle indicates methylation. In I-2 (grandmother), six subclones were successfully analysed, in the II-4 and III-3 eight subclones. All individual bisulphite sequencing data can be found in Figure 1 and Suppl. Table 1.

**Supplementary Figure 2**: Pedigree with results from the linkage analysis. The haplotypes are coloured, the red haplotype harbours the familial mutation. Upper right box shows the STR-marker names and position. The family’s T>C variant is localized between the second and third marker. There are crossovers in individuals III-3 and III-4. In individual II-4, the *marks a probable polymerase slippage.

**Supplementary Figure 3:** DNA methylation analysis: Heat maps of the CTS1 and CTS6 methylation patterns obtained by next generation bisulphite sequencing in normal (control) individuals without the OCT4 binding site mutation. The pedigree marks below the panels are the same as in Figure 1. Lines represent sequence reads, columns CpGs. Blue – unmethylated – maternal (mat); red –methylated – paternal (pat); white - missing sequence information.
